# Interpersonal Perception of Time-Use Patterns in Romantic Relationships: Protocol for the IP-COUPLES Study

**DOI:** 10.2196/21306

**Published:** 2021-05-04

**Authors:** Romain Bertrand, Brenda Vrkljan, Nicolas Kühne, Linda Charvoz, Nicolas Vuillerme

**Affiliations:** 1 Network "Human Occupation and Health" University of Applied Sciences and Arts Western Switzerland (HETSL | HES-SO) Lausanne Switzerland; 2 AGEIS University Grenoble Alpes Grenoble France; 3 School of Rehabilitation Science McMaster University Hamilton, ON Canada; 4 Faculty of Social Work University of Applied Sciences and Arts Western Switzerland (HETSL | HES-SO) Lausanne Switzerland; 5 French University Institute Paris France

**Keywords:** behavioral disciplines and activities, daily living activities, health, human activities, interpersonal relations, social interactions, spouses

## Abstract

**Background:**

Perceptual congruence has been defined as the level of agreement between partners on various aspects of their shared lives, including perceived engagement in individual and jointly performed activities. While the level of adjustment made by partners to such activities is thought to contribute to a couple’s sense of mutuality, perceptions of time use concerning activity engagement has yet to be considered. As such, this study will determine the level of perceptual congruence between partners with respect to perceived time use in their respective and shared activities.

**Objective:**

The primary objective of the IP-COUPLES study is to determine the similarities and differences between partners in terms of their perceptual congruence with respect to independent and jointly performed activities. This study will also examine the association between independent and joint activities in terms of perceptual congruence of time use and the strength of this association.

**Methods:**

This descriptive observational study includes 100 couples from Western Switzerland who are recruited using snowball sampling methods. The Life Balance Inventory (LBI), a self-report questionnaire that captures activity configuration congruence, will measure independent and joint perceptions of both time use allocated to daily activities and corresponding satisfaction. Due to COVID-19, the protocol can be administered virtually by the primary investigator. The mean scores of perceptual congruence variables will be used for analysis, namely perceived congruence of time use in terms of independent and jointly performed activities. For the first objective, an independent *t* test will be used for each variable to compare the mean score between activities on the LBI. For the second objective, the correlations between the mean scores for these activities will be calculated for each variable using the Pearson correlation.

**Results:**

The IP-COUPLES study protocol was developed in 2019 and 2020. Enrollment began in June 2020. Data collection will continue until October 2021 to account for time needed for recruitment due to the COVID-19 pandemic crisis. Analysis and presentation of results are expected in 2022.

**Conclusions:**

This study is exploratory, as it is the first to our knowledge to investigate how perceived time-use patterns with respect to independent or jointly performed activities are similar or different among romantic couples. By investigating the interpersonal perception of time-use patterns among couples, the IP-COUPLES study is an important first step to understanding how romantic partners’ daily activities are contributing to the level of satisfaction as a partner and as a couple and to the sense of mutuality between partners in a romantic relationship.

**International Registered Report Identifier (IRRID):**

DERR1-10.2196/21306

## Introduction

A romantic relationship has been described as a particular form of social interaction between 2 individuals where one of the aims is a mutually satisfactory relationship [1,2]. However, to achieve such satisfaction is a complex process, often requiring behavioral and psychological adjustments to ensure each partner’s respective needs and preferences are met in this relationship [3-5]. The notion of “we-ness” has been raised in social psychology in reference to a couple’s sense of mutuality. A sense of mutuality often emerges from shared time and experiences over the course of a relationship [6]. However, it is important to clarify that “we-ness” is an interpersonal entity that encompasses both partners [7]. Moreover, “we-ness” also reflects the reciprocity between partners and the ability to accurately and cogently consider the other partner’s perspective [8]. Not surprisingly, researchers have postulated couples with a high degree of “we-ness” are more likely to have a more satisfying relationship where the ability to adjust to one another’s needs is thought to be a contributing factor to satisfaction [8]. Romantic partners who are better able to connect with their partner’s respective experiences report higher rates of marital satisfaction [9,10]. In fact, such connectivity between partners is thought to support the unicity of the couple where patterns in their behavior and communication develop, as reflected in their shared activities or “patterns of doing.” While we expect shared ways of doing to be unique to each couple [11], it remains unclear as to how such patterns are reflected in a relationship. In other words, we have yet to fully understand “time use” in a coupled relationship, namely what activities are jointly done as a couple and what activities are independently done by each partner. We aim to further understand how romantic partners respectively and jointly perceive time allocated for independent and shared activities and the sense of satisfaction associated with such perceptions. Such research sets the stage for further study of how couples independently and jointly adjust their activities when navigating changes, such as the onset of medical conditions in one or both partners and the corresponding impact on the relationship and sense of “we-ness.”

### Time-Use Patterns Among Couples

Kaufmann, a French sociologist, argued that a coupled relationship emerges from the formulation of shared or coconstructed routines [1,2,12]. Hence, such routines are thought to be reflected in a couple’s time-use patterns. These patterns are defined as how people “spend and structure their time” within their everyday lives [13]. For those in coupled relationships, we expect time-use patterns to be reflected in both separate and joint activities [14-18]. Thus, everyday activities performed jointly as a couple are thought to contribute to the sense of unicity or mutuality of the relationship in question [11]. Mutuality between partners can also emerge when a partner adequately adjusts to the needs of the other partner, including those activities one does independently. In fact, each activity, whether independent or done jointly, must consider both the expectations and needs of each partner. Hence, a romantic relationship can require the synchronization of time-use patterns and corresponding activities between partners [13,19]. However, each partner may have a different perspective when it comes to synchronization and the time allocated to such activities. Each partner may have to adjust to the needs of the other partner in terms of the time allocated for particular activities, while also considering his or her own needs.

Previous research examining time-use patterns among romantic couples suggested such patterns can either positively or negatively impact a relationship. For instance, it has been suggested that the time spent together as a couple has a direct influence on the perceived quality of the romantic relationship [18,20]. Joint or collaborative engagement in daily activities, especially those that involve new experiences, have been shown to contribute to the well-being of respective partners [17,19,21] as well as feelings of mutuality as a couple [13,22,23]. Some researchers have suggested couples should spend more time on joint activities [17,19], particularly those activities that are more social or leisure in nature [13,17,19]. Based on the analysis of time diaries of 4043 Belgian couples, Glorieux et al [17] reported that couples spent approximately 53% of their total time together with no significant differences between couples who were married and unmarried, although no information was provided about the duration of the relationship. Sleeping, eating, and watching television were the most commonly identified joint activities. Interestingly, shopping and leisure activities were largely conducted independently. Most shared time was spent in the home, during meals, evenings, and the weekends [17]. Genadek et al [18] found female same-sex couples spent more time on joint activities compared to both heterosexual and male same-sex couples. These results suggest time spent on joint activities can influence the quality of the relationship, which may also correspond to perceived mutuality and to “we-ness.” Thus, when partners synchronize their time-use patterns, such synchronization requires each partner to allocate enough time for the other partner’s needs for independent activities as well as jointly performed (couple) activities. While it is thought that a couple's mutuality can be strengthened when a partner shares a similar perception in terms of these activities, we do not in fact know the impact of perceptual congruence with respect to time use on their relationship. Hence, examining and understanding similarities and differences in perceived time-use patterns between partners with regard to activities is important given what is known about the impact of time use on relationship quality [13,19,22]. Many studies of time-use patterns [24-26] have considered individuals as singular entities in terms of analyzing their everyday activities when in fact, daily life, for those in partnered (coupled) relationships, requires a complex interplay between individuals and their respective patterns of engagement. Hence, this study will further our understanding of the similarities and differences between partners in terms of their perceived time use when it comes to their independent and joint activities. The study design and methods for the IP-COUPLES study (Interpersonal Perception of time-use patterns among COUPLES) are based, in part, on the paradigm of interpersonal perception (IP).

### Interpersonal Perception (IP): Measuring Perceptual Congruence of Time-Use Patterns Among Couples

IP is defined in social psychology as “reciprocal perceptions” between individuals with regard to various topics, such as affect [27], feelings [28], food preferences [5], job satisfaction, or political opinions [28], between at least two individuals and the degree of congruence between these perceptions [29,30]. Perceptual congruence refers to the degree of agreement between partners’ perceptions [31]. It is “…the association between partners’ perception of one another” [32]. These perceptions are crucial for the relationship [5,32]. As such, the more partners are congruent in their perceptions of the other partner’s activities — for instance, they are able to perceive likes or dislikes in terms of time allocation — the higher their level of mutuality and satisfaction with the relationship [8,11,31,32]. Studies have also suggested perceptual congruence between partners could be an indicator of problems in the relationship. For instance, each partner has a high degree of accuracy in terms of identifying the perceived needs of their respective partner, yet they are not able to meet these needs, thereby leading to lower rates of marital satisfaction [5,28].

**Figure 1:**
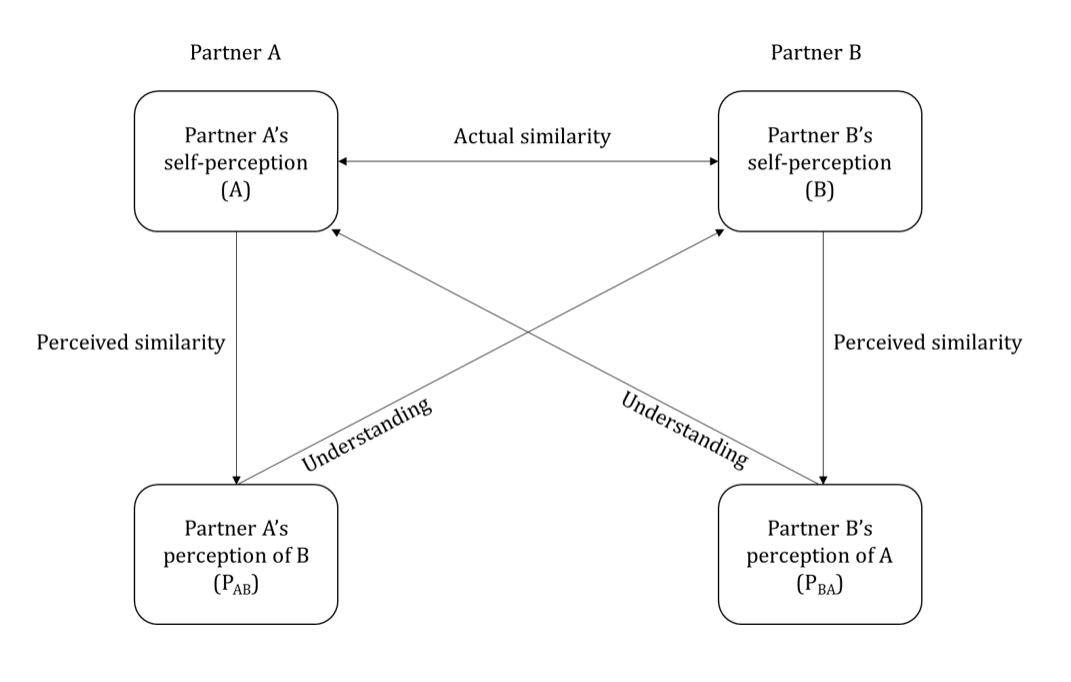
Reproduction of the model of perceptual congruence from Acitelli et al [33], presenting the 3 variables of perceptual congruence between partners in a romantic relationship. Arrows do not indicate a causal link but a correlation.

Acitelli et al [33] were among the first to propose a model (see Figure 1) to measure perceptual congruence in a romantic relationship. They identified 3 key variables of perceptual congruence. The first is “perceived similarity,” that is, the congruence between a partner’s self-perception and his or her perception of the other partner, where one partner’s own needs are projected onto the other partner [30]. In this way, there is assumed similarity, which refers to how one partner views the needs of the other partner as similar to oneself and thus, influences his or her perception of the respective partner. The second is “actual similarity,” which refers to the actual congruence between each partner’s self-perception, and the third is “understanding,” which refers to the level of congruence between a partner’s perception of the other partner and how the partner in question actually perceives himself or herself.

Using this model, studies of romantic couples have identified a significant link between the level of perceptual congruence and dyadic coping [34]. Dyadic coping of a couple is described as the level of interdependence required to address an external stressor. When one partner is experiencing distress, a response is often expected from the other partner. Research indicates that strong perceptual congruence between partners in their dyadic coping strategies are related to a partner’s respective level of satisfaction in the relationship [32].

Using the model put forward by Acitelli et al [33], the current study aims to build on our understanding of perceptual congruence among romantic couples by exploring the link between perceived time-use patterns in terms of individual and shared activities and the sense of mutuality in the relationship. For this purpose, we operationalize time-use patterns using the Life Balance Model [35,36]. In this model, life balance is defined as the configuration of time allocated to activities that are “healthful, meaningful, and sustainable to an individual within the context of his or her current life circumstances” [20]. The key component of the Life Balance Model is activity configuration congruence (ACC). ACC emerges from time-use patterns, where both the amount of time and corresponding satisfaction (with the time) allocated to daily activities are considered. Optimally, ACC reflects a balance between “one’s actual activity configuration in everyday life” and “one’s desired activity configuration in everyday life” [37].

The aim of the IP-COUPLES study is to examine perceptual congruence of ACC between partners in a romantic relationship. More specifically, this study will examine the perceived ACC of “independent” activities, as reflected by each partner’s ACC score on the Life Balance Inventory (LBI). Once this measure is completed, each partner will then complete his or her perceived ACC of “joint” activities. Finally, the couple will complete the LBI measure together. Consequently, this study will capture the following: (1) how each partner perceives his or her own ACC in relation to his or her own “independent” activities (LBI completed without the other partner), (2) how each partner perceives the ACC of his or her partner’s activities that are performed independently (LBI completed without the other partner), (3) how each partner perceives the ACC of joint activities that are performed together as a couple (LBI completed without the other partner), and finally, (4) how each couple jointly perceives ACC of their jointly performed activities (LBI completed together as a couple).

We expect a sense of mutuality to be reflected in the level of perceived congruence between partners in terms of engagement in both independent and joint activities [15,17]. While it is thought that each partner in a romantic relationship must synchronize their time-use patterns to meet each other's needs, it remains unclear if and how perceptions of time use between partners are similar or different from one another as well as how these patterns are perceived as a couple. From our results, we will also determine the association between independent and joint activities in terms of perceptual congruence and the strength of this association. In fact, results may emphasize a need to distinguish between independent and joint activities when designing interventions that address perceived synchronization of time-use patterns between partners. The current study sets the stage for future research focusing on the effect of potential interventions on time-use patterns and mutuality or “we-ness.”

### Objectives of the Study

The aim of the IP-COUPLES study is to examine the perceptual congruence of ACC among partners that are in a romantic relationship. The primary objective of the IP-COUPLES study is to determine the similarities and differences between partners in terms of their perceptual congruence with regard to time use in both independent and joint activities. As well, this study will examine the association between independent and joint activities in terms of perceptual congruence between partners as well as the strength of this association.

## Methods

### Study Design

This protocol involves a descriptive observational study that will be undertaken in Western Switzerland. This methodology is observational, meaning the focus is on exploring a specific phenomenon at a given point in time, namely perceptual congruence within romantic couples. Participant recruitment began in July 2020, and the aim is to finish data collection by October 2021.

### Sample and Recruitment

Previous studies on the notion of IP in coupled relationships were reviewed to determine the sample size necessary to achieve our intended objectives. To the best of our knowledge, no published studies have investigated time-use patterns in relation to the paradigm of interpersonal perception. We are aware that significant conclusions cannot be drawn due to the expected effect size. A post hoc calculation will be done to counterbalance this limitation. Kenny and Acitelli [28] included 238 married and unmarried couples to measure their perceptions with respect to well-being: feelings of closeness, feelings of caring, equity, enjoyment of sex, and job satisfaction. They calculated the correlation between the partners’ actual feelings. The coefficients ranged from 0.47 (job satisfaction) to 0.20 (equity). Vanderbleek et al [38] explored the correlation between couple play and couple satisfaction and stability. From 30 couples, they found coefficients of correlation of 0.70 (*P*<.01) between couple play assessment (CPA) and the satisfaction scale, 0.69 (*P*<.01) between CPA and the communication scale, 0.65 (*P*<.01) between the CPA and the conflict resolution scale, and 0.52 (*P*<.01) between the CPA and the idealistic distortion scale. Finally, Tucker and Anders [39] included 61 undergraduate couples who were dating where they assessed each partner’s attachment style, feelings about the relationship, and perceptions of the other partner’s feeling about the relationship. The coefficients of correlation for each partner’s perceptions of the other partner’s feelings about the relationship ranged from a mean of 0.31 (*P<*.001) for men to a mean of 0.41 (*P*<.001) for women. From selected studies, we determined our sample size using Pearson correlation calculations. We calculated a conventional large effect size of 0.5 (*P*<.05). Using the GPower software [40], we determined a sample size of 180 participants or 90 couples. Hence, the current study aims to recruit 100 couples, which is 200 participants in total. The recruitment of an additional 10 couples accounts for potential attrition of participants. Applying a post hoc power analysis on this sample size, a size effect of 0.5 (*P*<.05) gives a power value of 96%. Hence, this sample size is large enough to confirm our hypothesized effect size. Because of difficulties of recruitment due to the COVID-19 pandemic, our plan is to conduct an intermediary analysis. For this analysis, we aim to have 72 couples (144 individuals) to undertake a post hoc calculated power value of 90%.

Participation in this study is voluntary. Western Switzerland is a French-speaking region, which is the primary investigator’s native language. The choice to focus our sampling to this country is mainly due to the restrictions in place due to the COVID-19 pandemic. While we recognize limiting our sample size to this geographic region has consequences on the generalizability of our results, ensuring the contextual elements are similar is important. For example, public health measures in place for this region are likely to affect time-use patterns and activity engagement, and we expect these to be similar for the sample. For participant recruitment, announcements have been published in local newspapers, in e-bulletins, and on websites of associations targeting those who are retired, as well as sports- or cultural-related associations. If necessary, advertisements will be placed in the professional networks of the primary investigator for snowball sampling, which are people who work in health-related fields, such as occupational therapy and social work. The advertisement outlines the title of the study, its objectives, the inclusion criteria, the implications for participants, and how the results will be used. Details are also provided about how to contact the main investigator (RB). Couples who agree to participate in this study contact this investigator by phone or email in accordance with their preference. A brief overview of the study is then provided verbally as well as in writing, including ethical procedures. Inclusion, exclusion, and dropout criteria are reviewed at this time. Couples in which one or both partners require assistance in daily activities are excluded from the current study. The need for assistance may pre-suppose a health issue that could mean that one or both partners are more vulnerable, which can impact the dynamics of the relationship. Ensuring participants are protected from COVID-19 has been considered in the study design. Web-based meetings are strongly encouraged with the main investigator (RB). Finally, informed consent is sent by post or email in accordance with the participants’ wishes. Both partners are required to sign the consent form and return a copy to the main investigator.

### Inclusion Criteria

The inclusion criteria are cohabiting coupled partners, married and unmarried, where each partner is 18 years or older at time of data collection; the respective partners must consider themselves to be in a romantic relationship; the 2 partners read, understand, and speak French; the partners have lived together in the same residence for at least 1 year; and the couple lives in Western Switzerland at the time of data collection.

### Exclusion Criteria

The exclusion criteria are at least one partner has a disease or medical condition that requires assistance of the other partner or another caregiver with daily activities, at least one partner is under legal guardianship, and at least one partner does not give his or her consent to participate in the study.

### Dropout Criteria

The dropout criteria are that partners are not able to physically separate from each other during the meeting (eg, move to another room) and therefore can hear each other's responses to the questionnaire, partners exchange answers during the course of data collection, worsening of a partner’s health condition that requires the assistance of the other partner or caregiver with daily activities, and at least one partner revokes consent to the study.

### Data Collection

Data collection is completed by the first author of the study. Participants are given 2 options with regard to the location for data collection. Originally, the study was designed for an in-person, face-to-face meeting at a physical location chosen by the couple [28]. Because of the COVID-19 pandemic, a videoconference platform (eg, Zoom) is being offered as an option. This virtual alternative prevents a physical meeting with people who may be at risk for COVID-19 or for whom it is impossible to do the meeting outside and safely.

The main questionnaire used in this study is the LBI [35]. The LBI was developed by Matuska, based on the Life Balance Model [37]. It measures the life balance of individuals with respect to time allocated for different daily activities and their level of satisfaction with how their time is allocated for such activities. The LBI tracks time allocation across 53 activities (eg, shopping, driving, participating in groups, relaxing, participating in outdoor activities, working, using a computer, taking care of oneself, playing music, reading). For each activity, participants are asked to indicate yes if they do or if they want to do the activity in question. A participant will indicate no if they do not do or if they do not want to do the activity. If they answer yes, participants are then asked to rate, using a Likert scale, if they are able to spend the amount of time they desire on the activity where “1” indicates less time than desired (ie, “always less than what I want”) and “5” indicates more time than desired (ie, “always more than what I want”). The French version of the LBI has been validated [41] and will be used for the current study.

For the purpose of this study, the main investigator (RB) reads each question on the LBI and then lists the different options for participants to respond. To limit loss of data or misunderstanding, participants also have a printed copy of the questionnaire, so they can also read the questions and provide the answers as the questionnaire is administered by the investigator. The order of administration of the LBI is decided by partners at the outset of the initial meeting with the investigator, with one of the partners volunteering to go first. The partner who volunteers to start stays with the investigator (online), while the other partner moves far enough away, preferably to another room where he or she cannot hear any parts of questionnaires as they are administered. If there is no possibility to move to another room, the other partner will be asked to wear headphones (and to listen music if possible) so the sound is muffled.

The first partner to be administered the questionnaires will provide his or her sociodemographic information (eg, his or her age, education). At the second step, he or she completes the LBI a total of 3 times, using a different perspective each time: (1) self-assessment of his or her own ACC for activities that are independently performed, (2) his or her perceptions of how he or she thinks his or her partner would respond with respect to his or her own ACC for these activities, and (3) his or her perception of how he or she might answer when both partners are concurrently responding to the questionnaire concerning the perception of ACC for joint activities. When the first partner completes the LBI from these 3 perspectives, the other partner then enters the room and follows the same steps. The other partner is also asked to leave the vicinity so as not to overhear administration and responses. In the final step, both partners are brought back together to complete the ACC jointly as a couple. They will also provide some further information about their relationship at this final step: the length of their relationship, the number of children they have together, the ages of the children, and how many children are still living with them.

If a couple withdraws or cannot complete any step of data collection, their data will not be included in the final analysis. As per previous studies, questionnaires will be completed by each partner individually to avoid any discussion between partners concerning their perceptual congruence on any of the activities [28,42].

### Statistical Analysis

Statistics will be calculated using IBM SPSS version 25 [43]. For data coding, each couple will randomly be allocated a number. Partners in the couple will also be randomly allocated a letter, “A” or “B.” The couple will be referred to as “C.” For instance, we will refer to couple “1” as A1, B1, and C1; couple “2” as A2, B2, and C2; and so on. Prior to the analysis, the sample will be first described. The scores of LBI will then be reported as measures for the analysis that will aim to answer the study objectives. We will then do all calculations with the 3 variables of perceptual congruence, as described in the model by Acitelli et al [33]: (1) actual similarity, (2) perceived similarity, and (3) understanding of time-use patterns for activities performed independently and jointly, respectively. Because we expect to have a normally distributed sample, we will do parametric statistical tests.

### Analysis

Prior to the analysis, the sample will first be analyzed in terms of their descriptive statistics. Intra- and interindividual central tendency and dispersion of scores will be calculated for (1) each partner’s self-perception of his or her ACC for independent activities, (2) each partner’s perception of the other partner’s ACC for independent activities, (3) each partner’s perception of the ACC for joint activities, and (4) each couple’s perception of ACC for joint activities.

**Figure 2:**
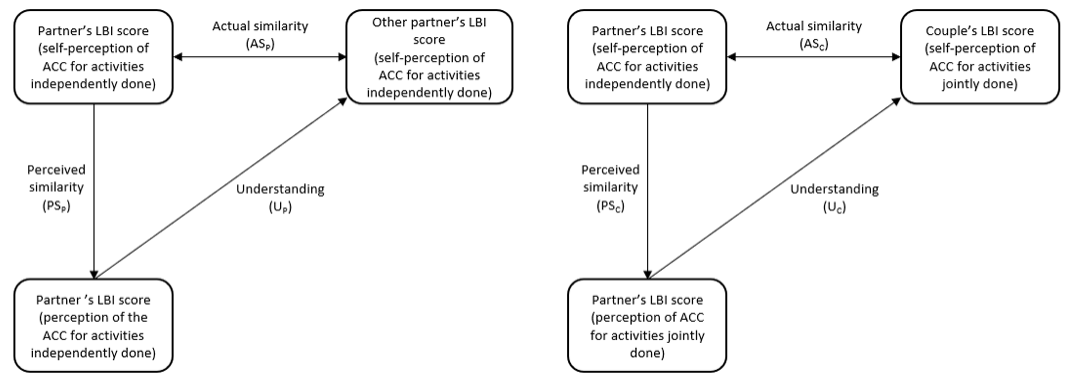
Coefficients of perceptual congruence of time-use patterns among couples that are used for statistical calculations (adapted from the model of Acitelli et al [33]). ACC: activity configuration congruence; LBI: Life Balance Inventory.

From the LBI scores, we will determine the different coefficients of the 3 variables that comprise perceptual congruence, as per Actelli et al [33] and the LBI (see Figure 2). Actual similarity between partners (AS_P_) is the ratio between 1 partner’s self-perception of ACC for his or her activities independently done and the other partner’s self-perception of ACC for his or her activities independently done. The actual similarity between one partner and his or her partner (AS_C_) is the ratio between one partner’s self-perception of ACC for his or her activities independently done and the couple’s self-perception of ACC for activities jointly done by partners. Perceived similarity between partners (PS_P_) is the ratio between one partner’s self-perception of ACC for his or her activities independently done and his or her perception of the other partner’s ACC for his or her activities independently done. Perceived similarity between one partner and his or her couple (PS_C_) is the ratio between one partner’s self-perception of ACC for his or her activities independently done and his or her perception of the of ACC for activities jointly done by partners. Understanding between partners (U_P_) is the ratio between one partner’s perception of the other partner’s ACC for his or her activities independently done and the other partner’s self-perception of ACC for his or her activities independently done. Understanding between one partner and his or her couple (U_C_) is the ratio between each partner’s perception of ACC for activities jointly done by partners and the couple’s self-perception of ACC for activities jointly done by partners.

The first objective of the IP-COUPLES study is to determine if there are similarities and differences between partners’ perceptual congruence of their independent and joint activities. The mean score of each variable of perceptual congruence will be calculated (1) between partners (AS_P_, PS_P_, and U_P_) and (2) between each person and his or her partner (AS_C_, PS_C_, and U_C_). Comparisons of mean scores will then be done using an independent *t* test: between AS_P_ and AS_C_, between PS_P_ and PS_C_, and finally, between U_P_ and U_C_.

The second objective is to examine the association between independent and joint activities in terms of perceptual congruence and the strength of this association. Correlations will be calculated for each variable of perceptual congruence using Pearson correlation. Three correlations will be calculated: between AS_P_ and AS_C_, between PS_P_ and PS_C_, and between U_P_ and U_C_.

### Data Protection

All collected data will be anonymized. They will be kept for 10 years, in accordance with Swiss recommendations specific to data storage [44]. Data are stored on an encrypted external hard drive of the main investigator disconnected from any network. The data are only shared between members of the authorship team. No specific information is provided to participants nor between partners. They do not receive any analysis of their respective relationship. They can only access the final version of the study where the results are consolidated. The cantonal commission of ethics for research on humans gave its approval for the project (protocol number 2019-00847).

## Results

The IP-COUPLES study protocol was developed in 2019 and 2020. Enrollment began in June 2020. Data collection will continue until March 2021, with ongoing adaptations due to the evolving COVID-19 pandemic crisis. Analysis and presentation of results are expected to be available in early 2022.

### Prior Analysis

First, the sociodemographic description of the sample will be reported in table format. To facilitate readability, information that concerns all partners as individuals and couples will be presented in 2 tables. A bar graph will then be used to show the central tendency and dispersion of participant scores concerning (1) each partner’s self-perception of his or her ACC for his or her independent activities, (2) each partner’s perception of the other partner’s ACC for his or her independent activities, (3) each partner’s perception of the ACC for joint activities, and (4) each couple’s perception of ACC for their joint activities. Coefficients of perceptual congruence variables will be presented in 2 tables. The first table will show the coefficients of perceptual congruence between partners’ perceptions of ACC for independently done activities, and the second table will present coefficients of perceptual congruence between partners’ perceptions of ACC for activities jointly done as a couple and those of the couple.

### Study Objectives

Each study objective will be addressed in a separate table displaying the respective results.

## Discussion

The current study aims to enhance our understanding of the relationship between mutuality of romantic couples and time spent (ie, time-use patterns), either independently or jointly, on performing everyday activities. This study is exploratory in nature, as it is the first to our knowledge to investigate how time-use patterns of couples and corresponding activities, whether independently or jointly performed, are similarly or differently perceived among partners in a romantic relationship. As previously noted, time-use patterns reflect couples’ ways of doing, which contribute to the unicity of the couple. Findings from previous dyadic studies suggest interacting in daily life as a romantic couple affects the level of interdependence, which may be reflected in the activities in which partners engage [15,16]. In other words, the needs of each partner can influence the other’s engagement in everyday activities. As such, the way partners perceive their respective partner’s level of engagement in daily activities can influence how much they adjust to meet the needs of their partner. In some cases, they may even sacrifice their own needs in terms of their activities to accommodate the needs of their partner. Hence, the degree of adjustment or accommodation has been raised in previous research where the willingness of partners to adjust to each other's needs in terms of activities is thought to strengthen the sense of mutuality experienced by the couple [8,11,31,32]. The current study seeks to further understand the role of perceptual congruence of each partner in terms of engagement in these different types of activities, which remains unclear.

By investigating the interpersonal perception of time-use patterns within couples, the IP-COUPLES study will make an important contribution as to how romantic partners’ daily activities contribute to feelings of satisfaction as a partner and as a couple and, in turn, the sense of mutuality between partners. By leveraging existing research on perceptual congruence and its related variables, we will be able to discern similarities and differences in how activities that are independently and jointly performed are perceived. Furthermore, we will go one step further in determining the extent to which each of these perceptions are related. We will also consider if there are significant differences between each variable of perceptual congruence, namely actual similarity, perceived similarity, and understanding [33].

This research sets the stage for future investigations that delve further into the perception of time-use patterns among couples. A next step in the IP-COUPLES study is to investigate the extent to which health-related changes in one or both partners can influence how daily activities are perceived by couples and, in turn, how it may influence feelings of mutuality between partners. Another study emerging from IP-COUPLES could subsequently investigate relationships between marital satisfaction and the degree of mutuality among couples and congruence with each partner’s perceptions of time-use patterns as a couple.

The current study should be considered in light of certain limitations. The LBI questionnaire was designed for use by a single participant and not for joint responses as a couple per se. Given there are no questionnaires currently designed to capture joint activities and time use, including satisfaction with such time use, the LBI is the best tool available to be used for this purpose. Hence, our research team will carefully track and record any challenges that arise with regard to administering the LBI in this way. We expect our study will highlight the need to design and validate questionnaires that can be administered in this way, given what is known about co-performance of everyday activities.

Another potential limitation is related to the COVID-19 pandemic. As a result of the pandemic and public health recommendations for social distancing, a large part of the world's population has been affected by changes in their daily activity patterns. In Switzerland, since March 2020, there has been alternating periods of public restrictions. Hence, responses by participants on the LBI may depend on the restrictions at the time of the interview. However, the focus of the current study is not so much on the activities, as it is on perceptual congruence. Nevertheless, partners may not be aware, or may be even more aware, of one another’s activity patterns and engagement. As previously noted, couples recruited for this study are expected to be from the same geographic region in Switzerland. While this sampling approach limits the generalizability of the findings, similar public health measures are expected to be in place in this region. Continuing to collect data and track participants in the IP-COUPLES study with regard to navigating the current pandemic and the postpandemic period is being considered by the study team.

## Abbreviations

ACC: activity configuration congruence
CPA: couple play assessment
IP: interpersonal perception
LBI: Life Balance Inventory
